# Letter to the Editor From Landrigan et al: “Chemicals Used in Plastic Materials: An Estimate of the Attributable Disease Burden and Costs in the United States”

**DOI:** 10.1210/jendso/bvae082

**Published:** 2024-04-23

**Authors:** Philip J Landrigan, Maureen Cropper, Sarah Dunlop, Yongjoon Park, Christos Symeonides

**Affiliations:** Global Observatory on Planetary Health, Boston College, Chestnut Hill, MA 02467, USA; Centre Scientifique de Monaco, Medical Biology Department, MC 98000, Monaco; University of Maryland, Economics Department, University of Maryland, College Park 20742, USA; Minderoo Foundation, Nedlands, WA 6009, Australia; The University of Western Australia, School of Biological Sciences, Perth, WA 6009, Australia; Department of Resource Economics College of Social & Behavioral Sciences, University of Massachusetts at Amherst, Amherst, MA 01003, USA; Minderoo Foundation, Nedlands, WA 6009, Australia

We thank Trasande et al for their estimate of the disease burden and economic costs attributable to plastic-associated chemicals in the United States [1]. We also salute the Endocrine Society on your continuing leadership in advancing the science of endocrine disruption.

In their otherwise excellent paper [1], Trasande at al make 2 errors of fact in describing the Minderoo-Monaco Commission on Plastics and Human Health [2], on which we are authors. First, Trasande et al state incorrectly that our Commission assumed all Polybrominated diphenyl ethers, phthalates, and bisphenols in the United States are used in plastic manufacture. To the contrary, we state clearly in our report that “Estimates suggest that over 90% of exposure to these substances comes from plastics.” We based our calculations of disease burden and costs on that estimate.

Second, Trasande et al are incorrect in their claim that we propagated a mathematical error in estimating the size of the population affected by phthalate exposure. In fact, it was Trasande et al who themselves first published this incorrect estimate in Table 4 of their 2022 paper in which they erroneously combined 2 years’ data into1 [3]. In the initial publication of our Commission report [2], we uncritically included their overestimate in our analysis. However, when we undertook a detailed examination of the primary data on which the 2022 Trasande et al analysis was based, we discovered the inaccuracy in their findings. Accordingly, we immediately notified Trasande et al of their miscalculation and we published an erratum [4]. Our corrected estimate is that phthalates in plastic were responsible for 45 381 deaths in the United States in 2013 and for economic costs of US$245 billion [4].

The convergent findings of the Minderoo-Monaco Commission [2] and the Trasande papers, all of which show that toxic chemicals in plastics are responsible for substantial burdens of disease and large economic losses, despite their somewhat different methodologies [1, 3], underscore the urgent need for a strong and legally binding United Nations Global Plastics Treaty [5]. Key provisions of this treaty need to be a global cap on plastic production, strict regulation of all chemicals in plastics, and restrictions on the manufacture of single-use plastics [5].
